# Alcohol and the Human Body

**Published:** 1918-03-16

**Authors:** 


### The Hospital

The Workers' Newspaper of Administrative Medicine and Institutional Life, Administration, National Insurance and Health.

No. 1658, Vol. LXIII. SATURDAY, MARCH 16, 1918. PRICE ONE PENNY

### ALCOHOL AND THE HUMAN BODY.

The plain man may read with some surprise the statement that "throughout the world lack of exact knowledge still prevails about the action of alcohol on the human system." Yet the proposition rests on no less an authority than that of Lord D'Abernon, the Chairman of the Central Control Board (Liquor Traffic), and, as all the world knows, the Central Control Board has had to deal with the alcohol question during the war in a highly practical fashion. Naturally, therefore, the Board has been driven to seek some principles on which to base its advice and recommendations. Among various questions it has had to face is the effect of alcohol on industrial efficiency, and a full answer to this must obviously include a knowledge of the physiological action of alcohol. In the hope of obtaining this the Central Board appointed an Advisory Committee of eight medical men of high scientific standing, who were instructed to report on "the effects on health and industrial efficiency produced by the consumption of beverages of various alcoholic strengths," and to "plan out and direct such investigations as may appear desirable with a view to obtaining more exact data on this and cognate questions." The Advisory Committee duly met and soon decided that before further research could be surely guided to points of practical importance it was essential to draw up a brief statement of the present position of knowledge. The outcome of this decision is a small book of some 140 pages, named "Alcohol: its Action on the Human Organism," issued by H.M. Stationery Office, and to be obtained through any bookseller for a modest half-crown. Upon this book is based Lord D'Abernon's verdict that "lack of exact knowledge still prevails about the action of alcohol on the human system."

The first thought which such a judgment excites is surely that of the contrast between controversial activity and real knowledge. Here is a subject about which volume after volume has issued from the press, myriads of letters have been written to unnumbered editors, legions of orators have perorated from the platform, and pamphlets and discussions have multiplied themselves in thousands. Yet at the end of it all, when a Committee of detached, impartial, scientific men sit down to determine what is necessary for the further elucidation of the subject their first conclusion is that, while certain facts can be accepted as established, there are "numerous directions in which information concerning the action of alcohol is still so vague and unsatisfactory that no conclusion is legitimate." For various reasons the whole question, as is notorious, has been involved in controversy, and the partisans on one side and on the other have not unnaturally been more anxious for victory than for the establishment of scientific accuracy. Hence in the pursuit of this aim many propositions have been taken for granted which it now appears rest upon surmise and superficial observation rather than on demonstration and experiment. It is a decided gain amidst the mass of contradictory statements and accusations to have the result of a calm, reasoned investigation conducted for the mere purpose of discovering truth and of defining both what is known and what is unknown. When from such an investigation there issues the announcement that there are "numerous directions" on which actual knowledge is still vague and unsatisfactory, partisans may feel impatience at such hesitations, but those who desire to know the facts will be inclined to rejoice. There is further the broad lesson that confident words and actual knowledge are not necessarily one and the same thing. The one may have its platform triumphs, but only the latter has an abiding value. To many, a confession of ignorance has an appearance of weakness, but such is not the teaching of scientific method. Here rather it is the first boundary to be defined, for only after it has been defined can plans be formed for the extension of knowledge. This is the plan which has been adopted by the Advisory Committee, and their present Report is the first part of the scheme. Knowing exactly where knowledge fails they will next proceed to embark upon a plan of campaign for its enlargement, and the enterprise, it may be hoped, will be used as a model for the settlement of other issues. It is slow, it is unexciting, it lacks the attractive spice of controversy. But it is the only sure method of discovering the truth, and truth is essential to right action in regard to many issues on which the nation will ere long have to take a decision. Hence we welcome the Advisory Committee's Report, not only as an able and rigidly impartial statement on a much-debated question, but also because it indicates the penetration of scientific method into at least one of our governing and administrative organisations. Controversy has been loud and long. Now at last we get a stern presentation of the limits of actual knowledge.

One of the early lessons which is illustrated by the present Report is the necessity, as a condition of useful discussion, of the definition of terms to be used in the discussion. How often has time been lost and temper excited over the issue—"Is alcohol a food?" Or, again, on such a statement as—"Alcohol is a poison." Yet before setting to with their weapons the combatants have neglected to agree on the sense in which the essential words "food" and "poison" are to be severally understood. There are, of course, scores of similar examples in popular controversy, and we are not so simple as to imagine that the habit is going to be easily cured either by the present Report or by our personal exhortations. But the former at all events will probably be widely read, and apart from its conclusions it can hardly fail to have an educational value on the general public as an exhibition of the conditions essential to fruitful discussion. Our medical readers will hardly be surprised at any of the statements, positive or negative, made by the Committee, but they will find this small volume convenient as a succinct and authoritative presentation of what is really known on the subject of which it treats.

